# Medical Certificates for Life Insurance Offices

**Published:** 1851-03

**Authors:** 


### MEDICAL CERTIFICATES FOR LIFE INSURANCE OFFICES.

A topic that has for a long time been agitated in the British medical journals, is the adjustment of fees between Life Insurance offices and the medical practitioners who furnish certificates as to the health of persons proposed for insurance. The recent great increase of the business of life insurance in the United States, brings this question home to American practitioners, and calls for some allusion to it at our hands. For the benefit of such of our readers as have not been brought in relation with the subject, it may be well to observe, that, as a preliminary step to effecting an insurance on the life of an individual, the offices require a certificate of his eligibility, and an opinion as to his probable longevity from his medical attendant. This is, however, only an initiatory step, as before accepting any risk, the offices subject the applicant to an examination by their own medical adviser, whose opinion determines their decision in the case. The medical officer attached to the life insurance company, is of course paid by the office. But it has been contended, that the physician of the applicant should also receive compensation from the insurance company for the certificate which he is asked for. And this is the unsettled point—some physicians refusing to answer the schedule of questions proposed with reference to their patients, unless accompanied with a fee.

This is rather a question of a business than a professional character, and much may be urged upon both sides of the matter in dispute. It appears to us, however, (and this seems to be the opinion of such of our cotemporaries as have noticed the controversy,) that the weight of propriety inclines to holding the applicant for life insurance responsible for the remuneration of his own medical attendant. In all proposals for life insurance the basis of the contract between the party proposing and the office, is an unreserved communication of every fact in any way bearing upon the party's past and present condition of health. Naturally, the medical attendant of the applicant is looked to as the best means of supplying the required information, and it is on behalf of and as the organ of his patient, that he furnishes it; his opinion of the value of the life proposed for insurance not deciding the company in its final acceptance or rejection.

It has also been urged in some of the British journals, that if fees were indiscriminately paid by the offices, on presenting the schedule of questions to the applicant's medical referee, collusive applications might be tendered on lives, of the ineligibility of which no doubt could exist. This argument is, of course, inapplicable to the great body of the profession. But when we bear in mind the number of irregular practitioners, whose certificates may be received on these occasions, we cannot condemn the insurance companies for desiring to protect themselves against possible fraud.

